# Nanoscale Delivery Systems of Lutein: An Updated Review from a Pharmaceutical Perspective

**DOI:** 10.3390/pharmaceutics14091852

**Published:** 2022-09-02

**Authors:** Aslihan Hilal Algan, Ayca Gungor-Ak, Aysegul Karatas

**Affiliations:** 1 Department of Pharmaceutical Technology, Faculty of Pharmacy, Ankara University, Ankara 06560, Turkey; 2 Department of Pharmaceutical Technology, Faculty of Pharmacy, Bulent Ecevit University, Zonguldak 67600, Turkey

**Keywords:** lutein, xanthophylls, antioxidant, anti-inflammatory effect, degenerative diseases, drug delivery systems, nanoencapsulation

## Abstract

Carotenoids are natural lipid-soluble pigments that produce yellow to red colors in plants as well as providing bright coloration in vegetables and fruits. Lutein belongs to the xanthophyll subgroup of the carotenoid family, which plays an essential role in photosynthesis and photoprotection in nature. In the human body, lutein, together with its isomer zeaxanthin and its metabolite meso-zeaxanthin, accumulates in the macula of the eye retina, which is responsible for central, high-resolution, and color vision. As a bioactive phytochemical, lutein has essential physiological functions, providing photoprotection against damaging blue light, along with the neutralization of oxidants and the preservation of the structural and functional integrity of cellular membranes. As a potent antioxidant and anti-inflammatory agent, lutein unfortunately has a low bioavailability because of its lipophilicity and a low stability as a result of its conjugated double bonds. In order to enhance lutein stability and bioavailability and achieve its controlled delivery to a target, nanoscale delivery systems, which have great potential for the delivery of bioactive compounds, are starting to be employed. The current review highlights the advantages and innovations associated with incorporating lutein within promising nanoscale delivery systems, such as liposomes, nanoemulsions, polymer nanoparticles, and polymer–lipid hybrid nanoparticles, as well as their unique physiochemical properties.

## 1. Introduction

Phytochemicals are bioactive non-nutrient compounds found in grains, vegetables and fruits that are often associated with prevention of diverse diseases [1]. Among the more than thousands of phytochemicals that have been identified, phenolic compounds, alkaloids, organosulfides and carotenoids are the major phytochemical groups [2]. Producing yellow, orange, or red colors, the natural lipophilic pigments carotenoids provide a bright coloration in vegetables and fruits [3,4]. They are classified based on their chemical structure as either carotenes (hydrocarbon carotenoids) or xanthophylls (oxygen-containing carotenoids) [5,6]. Lutein and its structural isomer zeaxanthin are two major xanthophylls and are also called oxygenated carotenoids [7,8]. The xanthophylls lutein and zeaxanthin are yellowish-colored phytochemicals that are selectively concentrated in the macula of the eye retina after crossing the blood–retina barrier [9,10]. The macular region of the retina is yellow in color due to the presence of these macular pigments [11,12]. Of the more than 600 carotenoids present in nature, only lutein and zeaxanthin are situated in the macula lutea—namely, the ‘yellow spot’ [13,14]. Meso-zeaxanthin, known as the third xanthophyll, is a metabolite of lutein that is formed at the macula via metabolic transformation [15,16]. To protect the retina against oxidative damage and blue light, in nature, xanthophylls are used as efficient photoprotectors that can neutralize photosensitizers and reactive oxygen species (ROS) [17,18]. The macular pigment also protects sensitive photoreceptors from impairment induced by sunlight, indoor lighting, and even light emitted from electronic devices [19,20]. This protection helps to sustain healthy vision as well as to reduce the risk of aging- and inflammation-based eye disorders such as age-related macular degeneration (AMD) [21,22], diabetic retinopathy [23], and cataracts [24].

As a phytochemical, although lutein delivery seems to be an issue for food science and technology, the delivery of lutein is now also on the agenda of pharmaceutical technology, as it is also considered a bioactive ingredient due to its well-known antioxidant and anti-inflammatory activities [25,26]. Due to lutein’s importance for human health, its nutraceutical value comes to the forefront. The term nutraceutical, a *portmanteau* of the words “nutrition” and “pharmaceutical”, was originally coined by De Felice [27]. The nutraceuticals can be defined as “the phytocomplex if they derive from a food of vegetal origin, and as the pool of the secondary metabolites if they derive from a food of animal origin, concentrated and administered in the more suitable pharmaceutical form” [28]. In this novel concept, the therapeutic value, regulatory framework, efficacy, quality and safety issues of nutraceuticals have been clarified with the important contributions of many researchers [29,30,31,32,33,34]. Lutein has been frequently reviewed and discussed in the literature due to its therapeutic efficacy in disease prevention [35,36,37,38], but lutein delivery via novel drug delivery systems, especially within nanocarriers, has received limited attention [39,40]. For this reason, we have put together a review describing the techniques developed to maintain the stability and increase the effectiveness of lutein via nanoscale delivery systems, starting with a general introduction to its structure, biological functions, and therapeutic potential. In this current review, we offer a pharmaceutical perspective and focus on drug delivery strategies, paying particular attention to the success of nanoparticulate delivery systems.

## 2. Overview of Lutein

### 2.1. Structure and Biological Behavior of Lutein

**Figure 1:**
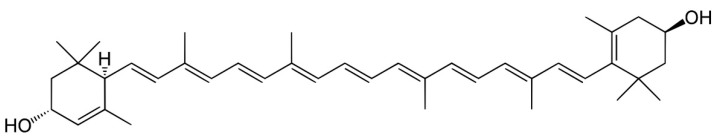
Structure of lutein.

The xanthophylls lutein and zeaxanthin are polyisoprenoids with cyclic structures at each end of their conjugated polyene chains that are highly reactive, with an electron-rich arrangement [41,42]. They both consist of elongated double-bond systems that create a light-absorbing chromophore, which is responsible for xanthophyll’s attractive color and special features [43]. Lutein and its isomer zeaxanthin structurally differ from each other in terms of the position of their double bond in only one of their rings [44]. In nature, the key feature that differentiates xanthophylls from other carotenoids (such as beta-carotene and lycopene) is their characteristic hydroxyl groups [45]. Lutein and zeaxanthin, unlike hydrocarbon carotenoids, have two hydroxyl groups, one at each end of the molecule, which are thought to play a crucial role in their biochemical functions and enable them to orient themselves inside cell membranes in a way that other carotenoids cannot [46,47]. Across the membrane, lutein and zeaxanthin orient their polar and nonpolar regions to align with the respective polar (protein) and non-polar (lipid) regions of phospholipid bilayers [48]. It has been proposed that xanthophylls’ unique intra-membrane orientation serves as “molecular rivets” that enhance the cell membrane’s intrinsic robustness while decreasing the vulnerability of membrane lipids to oxidation [49]. It has also been suggested that xanthophylls have more ability to resist autoxidation than other lipid-soluble carotenoids [50]. This physicochemical stability within the cell membrane improves their protective effects in various tissues [51]. The selective buildup of xanthophylls in the retina and neural tissues has been attributed to these distinctive traits, which have been identified throughout evolution [52]. Figure 1 shows the structure of lutein.

Currently, there is no recommended daily intake (RDI) or recommended dietary allowance (RDA) for lutein, but nutritionists advise consuming at least 6 mg of lutein per day for eye health. Especially for some population groups, a daily consumption dose of up to 10 mg of lutein is recommended for the treatment of AMD [53]. As lutein is naturally biosynthesized in plants and cannot be synthesized by human body, the primary source of lutein intake is through our diet or dietary supplements [54]. Dark green vegetables, including kale, spinach, parsley, broccoli, and turnip greens, are particularly rich in lutein. It also naturally occurs in yellow–orange foods such as corn and egg yolk [16,55]. The lutein molecule’s flexible and lipophilic nature boosts its affinity to lipid transporters, enhancing its absorption in the intestine [46,47]. It is thought that both simple diffusion and facilitated transport are involved in the absorption of lutein, and cholesterol membrane transporters such as scavenger receptor class B type 1 (SR-B1) play a significant role in this process. It is widely hypothesized that lutein follows the same absorption route as other lipids [56,57]. In the stomach, lutein is emulsified into tiny oil drops or globules and afterwards is incorporated into mixed micelles in the aqueous digestion fluid through the help of bile salts together with phospholipids. These micelles, promoting lutein accessibility during digestion by facilitating solubilization, are subsequently taken up by enterocytes via SR-BI. Following the passage through the intestinal epithelium, lutein is packed into chylomicrons and transported to various lipophilic tissues via lipoproteins through blood circulation. It then accumulates in adipose tissue, the liver, the brain, and particularly the eyes [37,58].

### 2.2. Therapeutic Potential of Lutein

Owing to lutein mainly being delivered to the retina, most studies have focused on its curative and protective effects on the eye [58,59]. Considerable research has been undertaken on the preventative effects of lutein on eye-related diseases, especially AMD and cataracts [60,61,62,63,64,65]. Given that lutein has potent antioxidant with anti-inflammatory properties, its undeniable efficacy against degenerative diseases has received great attention [66,67]. In the last few decades, a considerable number of studies have been conducted aiming to understand the effects of lutein on age-related and inflammation-induced diseases such as cancer [68,69,70,71,72,73,74,75,76,77], neurodegenerative disorders [78,79,80,81,82,83,84,85], cardiovascular diseases [86,87,88,89,90,91], and obesity [92,93,94,95,96]. In order to produce new knowledge on the benefits of lutein, its cytoprotective and disease prevention effects have been evaluated by extensive research focusing, in particular, on its mechanisms of action [97,98,99,100,101,102].

As is widely known, ROS are the pivotal molecules responsible for the harmful effects of oxidative stress, and free-radical-induced oxidative stress activates inflammatory mediators, leading to the development of many metabolic and degenerative diseases [103,104]. As a potent antioxidant, lutein quenches singlet oxygen and scavenges free radicals [22,105]. It reduces ROS production [106,107] as well as suppressing the expression of inducible nitric oxide synthase (iNOS) [108] and cyclooxygenase-2 (COX-2) [109]. In many studies, it has been revealed that lutein can modulate the inflammatory signaling pathways by the regulation of various molecular mechanisms that play a role in the inflammation process [67]. It suppresses the activation of pro-inflammatory cytokines such as tumor necrosis factor-α (TNF-α), interleukin-1β (IL-1β), and interleukin-6 (IL-6) [110,111]. Lutein also inhibits the activation of important cell-signaling pathways such as the transcription factor nuclear factor-kappaB (NF-κB), which modulates anti-inflammation and antioxidant defense [112,113,114]. Furthermore, it promotes the activation of nuclear factor erythroid 2-related factor2 (Nrf2), which is a regulator of cellular resistance to oxidants [83,115,116]. In addition, it enhances the expression of enzymatic antioxidants such as glutathione-S-transferase (GST), glutathione peroxidase (GPx), and catalase (CAT) together with the non-enzymatic antioxidant glutathione (GSH) [107,110]. By one or more of these mechanisms, it is quite possible that lutein may regulate immunological pathways, modulate inflammatory reactions, and alleviate oxidative damage. However, further research is required to confirm its main underlying molecular mechanisms of action.

## 3. Nanoscale Delivery Systems of Lutein

**Figure 2:**
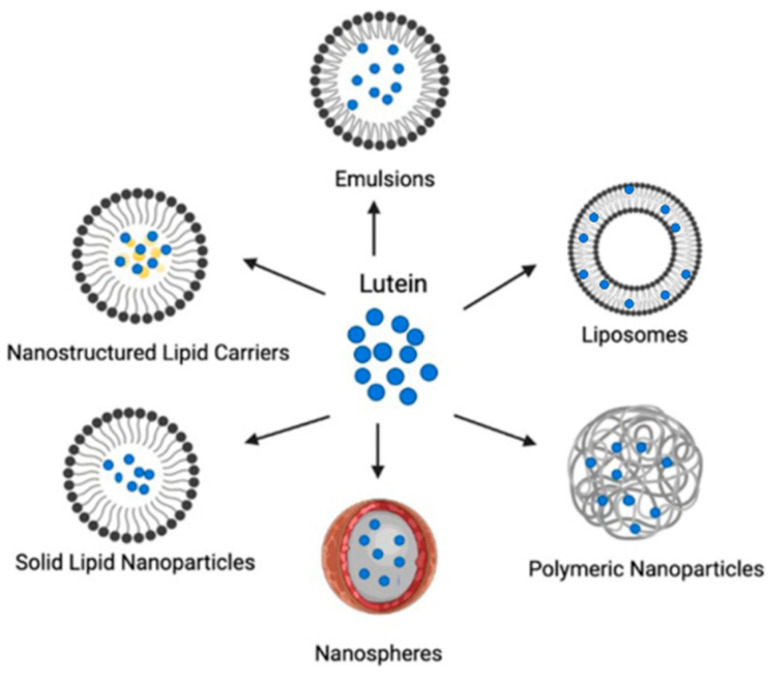
Schematic representation of nanoscale delivery systems for lutein.

Despite the therapeutic potential resulting from its diverse physiological activities, the effective delivery of lutein is difficult because of problems associated with its absorption and stability [67,117]. In order to overcome these limitations, researchers have examined several techniques used for maintaining the stability of lutein while enhancing its solubility and bioavailability [118]. In recent decades, nanotechnological applications on pharmaceuticals have been intensively studied. Just as with nanopharmaceuticals, nanotechnology has begun to be applied for superior delivery of nutraceuticals, thus a new class of nanomaterials, the nanonutraceuticals, has emerged [119,120,121]. Over the past few years, outstanding improvements have been made in the development of novel drug delivery systems for the encapsulation of bioactive ingredients [122]. For optimizing the delivery of lutein by enhancing its efficacy and stability, many vesicular and particulate drug carriers such as micelles, nanoemulsions, liposomes, and lipid- and polymer-based nanoparticles have been developed [123]. These delivery systems are prepared by employing various preparation techniques and characterized by features such as particle size, surface charge, encapsulation efficiency, and production yield. Some of these traits also govern their in vivo behavior [121]. Moreover, each delivery system possesses its own advantages and shortcomings. The efficacy of a delivery system is characterized by its biocompatibility, biodegradability, processability, loading capacity, and targeting ability [124]. Studies describing the success of lutein-loaded drug delivery systems are presented and reviewed below. Figure 2 provides a schematic representation of nanoscale delivery systems used for lutein.

### 3.1. Liposomes

Liposomes are vesicular structures composed of one or more lipid bilayers enclosing aqueous compartments in colloidal form [125]. They are generally 0.05–5.0 μm in diameter and take shape spontaneously when specific phospholipids are hydrated in aqueous media. They have the ability to carry water-soluble or non-soluble molecules due to the hydrophilic and hydrophobic regions they contain [126]. Hydrophilic compounds can be entrapped in the aqueous center of the liposomes, while hydrophobic compounds have more affinity for the lipophilic chains of the phospholipids that constitute the bilayers of the liposome [127]. Liposomes, acting as drug carriers, possess many advantages, such as a high drug loading capacity, spontaneous forming ability, and biocompatibility, along with a wide variety of physicochemical features that can be modified to control their biological attributes. With all these features, liposomes have emerged as effective carrier systems for the delivery of herbal constituents by improving their therapeutic efficiency and removing some of their limitations [128]. Entrapment within liposomal systems is able to conserve lutein effectively from early degradation and deactivation; thus, various liposome formulations have been developed and characterized with regard to their application in efficient lutein delivery [129].

In a series of studies that started with lutein encapsulation and continued with four kinds of carotenoids (lutein, β-carotene, lycopene, and canthaxanthin), a novel type of liposome system composed of mixed lipids including egg yolk, phosphatidylcholine, and non-ionic surfactant (Tween 80) was examined. The stability of carotenoids against the environmental challenges encountered during preparation, storage, heating, and surfactant dissolution was shown to be enhanced by liposome encapsulation. Additionally, it was shown that liposomes and carotenoids have a protective synergistic effect that is highly dependent on the molecular composition and concentration of the carotenoids. For example, it has been stated that carotenoids such as lutein that include oxygen-containing groups in their structure might be able to drastically alter the dynamics and organization of membranes [130,131,132,133]. It was also demonstrated that lutein encapsulation, particularly at higher concentrations, altered the liposome bilayer membrane’s physical characteristics, such as its phase transition temperature, lipid fluidity, polarity, and hydrophobicity [130,134]. Additionally, it was noted that the increasing concentration of encapsulated carotenoids most probably induced the outset of lipid pro-oxidation; thus, concentration should be optimized when producing liposomal systems containing carotenoids. Among carotenoids, lutein was reported to have the strongest antioxidant activity [134], the strongest ability to be incorporated into liposomes, and thus the highest loading capacity [132]. Likewise, lutein’s bioaccessibility was found to be strongly dependent on its ability to be incorporated into the lipid bilayer of liposomes [133].

For the lutein-loaded liposome systems produced by the supercritical carbon dioxide (SC-CO_2_) process, in one study, the supercritical anti-solvent (SAS) technique was used to create proliposomes including lutein and hydrogenated phosphatidylcholine. When proliposomes were hydrated, the lutein-loaded liposome dispersions formed spontaneously. The results indicated that lutein-loaded liposomes could be created easily by hydrating proliposomes with an average diameter of 200 nm and an encapsulation efficiency of 90.0%. It was demonstrated in this study that SAS technology could be utilized for manufacturing lutein proliposomes successfully [135]. In another study in which lutein-loaded liposomes were prepared by utilizing the SC-CO_2_ process, the lutein–lipid ratio was shown to have a serious impact on the size distribution and encapsulation efficiency, as well as on the morphology of the carriers. The use of the SC-CO_2_ method resulted in liposomes with superior characteristics, which were highly correlated with CO_2_ dispersion during the procedure. Due to the phospholipids and lutein being rearranged during the depressurization phase, it was shown that the encapsulation efficiency and placement of lutein within the phospholipid membrane were strongly reliant on the pressure used during their production. In this study, the mean dimensions of liposomes ranged between 148 nm and 195 nm, while the encapsulation efficiencies ranged between 57% and 97.8%. The researchers stated that this novel method offered great promise for use in loading a wide range of bioactives in liposomes and, especially, in scaling up production, instead of conventional methods [136]. In one of the latest studies on the SC-CO_2_ concept, the SuperLip technique, which promises a very high encapsulation efficiency, a low solvent residue, and a long-term stability of liposomes, was used by Trucillo et al. The liposomes developed in this study had mean diameters between 137 and 267 nm, and the encapsulation efficiency of lutein was as high as 98%. The researchers found that raising the production temperature boosted the drug release and hence reduced the release time, which was a predicted finding. It was shown in the study that by adjusting the manufacturing temperature as desired, the release of lutein could be controlled. As a consequence, it was stated that the manufacturing of these types of temperature-responsive liposomes utilizing high-pressure continuous manufacturing techniques may open up new avenues for understanding temperature-responsive vesicles [137].

On the other hand, it was shown that the physical stability of lutein-bearing liposomes could be enhanced by coating them with various biocompounds. In an earlier study, liposomes were coated using chitosan, a cationic biopolymer, and carotenoid-loaded nanoliposomes—namely, chitosomes—were obtained with particle sizes of 70–100 nm. It was shown that these could effectively bind to the liposomal surface, preventing lipid molecules from moving freely and maintaining the liposomes’ spherical form. The stability of lutein-loaded liposomes under a range of temperatures, gastrointestinal conditions, and levels of sedimentation during centrifugation was found to be directly increased by the hardening effects provided by chitosan. Additionally, the thin chitosan-coated layer favored the carotenoid encapsulation efficiency and maintained the monodispersion of the liposomes. Furthermore, it was explained that the carotenoids are retained in liposomes to different degrees depending on their molecular structure, with lutein and β-carotene being more effectively sheltered than canthaxanthin and lycopene [138]. In a later study, a cationic polypeptide, poly-L-lysine (PLL), was also utilized for modifying and protecting lutein-loaded liposomes. PLL-decorated nanoliposomes increased not only the stability, but also the release and bioactivity of lutein. The particle size of PLL-coated lutein nanoliposomes was determined to be between 264 and 367 nm, and the prepared structures were shown to be able to protect lutein effectively from gastrointestinal fluid conditions and degradation. Likewise, it was observed that the PLL-coated nanoliposomes increased the antioxidant and antiproliferative activity of lutein. In addition, compared to uncoated lutein nanoliposomes (68%) and free lutein (84%), the PLL-coated lutein nanoliposomes (60%) were shown to significantly increase the inhibitory activity against Caco-2 colon cancer cells. This was possibly because the PLL coating augmented the permeability of the cell membrane, thereby increasing the cellular absorption. It was stated in the study that the PLL-coated lutein-loaded nanoliposomes could be used as a promising system for increasing the bioavailability and efficient transport of lutein [139].

### 3.2. Emulsion-Based Systems

As mentioned in the introduction, lutein has lipophilic characteristics and a very low water solubility, limiting its direct inclusion in water-based compounds. As lutein’s chemical structure is unstable in acidic environments and very vulnerable to oxidative deterioration in the presence of light or heat, this kind of external factor can cause its easy degradation and reduce its bioactivity [33]. Due to its low stability in the gastrointestinal tract in the presence of the food matrix, digestive enzymes, and the strongly acidic environment, only a small proportion of lutein is absorbed, resulting in its low bioavailability [140]. For this reason, one of the effective formulation approaches which have been developed to encapsulate, protect, and deliver lutein efficiently is emulsion-based systems [141]. Emulsion-based delivery systems can be successfully prepared using oil phases, surfactants, and co-surfactants, and these systems have been extensively studied in pharmaceutical research with regard to their ability to encapsulate lipophilic drugs and enhance their bioavailability in the body [142,143,144].

In an earlier study, which was also the sole human clinical study investigating the nanodelivery of lutein, a lutein-loaded oil-in-water (O/W) emulsion system was prepared and converted into a nanoemulsion using the Microfluidizer^®^ Processor and compared with lutein supplement pills with regard to its bioavailability. A lutein nanoemulsion presenting 150 nm droplets on average showed a considerably greater bioavailability even at 10–40% lower doses than the lutein supplement, evidencing that nanoemulsion systems could improve the bioavailability of lutein even at physiological doses. After oral medicament, the bioavailability of lutein was found to be 62% higher than that obtained after the supplements were given, emphasizing that nanoemulsions could be utilized for the effective oral administration of lutein [145].

In another study, an oil-in-water (O/W) emulsion was prepared as a lutein carrier system using the ultrasonication method. In a study in which the emulsifier selection was investigated with regard to effective emulsion formation, six different emulsifier compositions (Tween 20, Tween 20/lecithin, Biozate 1, Biozate 1/lecithin, β-lactoglobulin, and β-lactoglobulin/lecithin) were evaluated. The effects of the emulsifier used were investigated in terms of the droplet size and resistance of the nanoemulsion system, the stability of lutein, and the uptake of lutein within human colon carcinoma cells (HT29). Due to their small globule size and high endurance to creaming, which are both signs of physical stability, it was revealed that the emulsions formed of biozate-1 and β-lactoglobulin in combination with lecithin individually demonstrated the most favorable results. Additionally, the formulation produced by these emulsifiers protected lutein from oxidation. All the results indicated that the type of emulsifier used strongly affected the cellular uptake, and the highest uptake was obtained with the use of a Biozate 1 and lecithin combination as an emulsifier system, which led to smaller droplets [146].

In order to investigate the bioavailability as well as the effects of lutein nanoemulsion on metabolic parameters in the plasma, liver, and adipose tissue, a hepatic steatosis model based on guinea pigs was created. In this study, an oil-in-water lutein nanoemulsion with an average droplet size of 254.2 nm was prepared using lutein powder, medium-chain triglyceride, d-a-tocopheryl polyethylene glycol succinate, and water. As compared to lutein powder, the lutein nanoemulsion increased the level of lutein in the liver and plasma by 1.6- and 2-fold, respectively. The results displayed a 55% reduction in oxidative stress in the lutein emulsion treatment group (3.5 mg/day) when compared to the control group after a six-week period (*p* < 0.05). In the lutein nanoemulsion treatment group, the hepatic steatosis score, hepatic triglycerides, and IL-6 levels were found to be significantly lowered. On the other hand, a small increase in the levels of plasma triglycerides, high-density lipoprotein (HDL) cholesterol, and low-density lipoprotein (LDL) cholesterol was observed, and the effects of the emulsion on the plasma lipids profile were attributed to the presence of a large number of medium-chain triglycerides in the formulation. It was indicated in this study that lutein nanoemulsion has protective effects on hepatic steatosis, despite it having several negative effects on cholesterol levels [147].

As local drug delivery vehicles, nanoemulsion (NE) systems serve as promising tools for ophthalmic application because of their ability to better wet, spread, and penetrate the inner layers of ocular tissues, as well as having sustained release ability [148,149]. Regarding ophthalmic lutein delivery, in one study, an NE system consisted of triacetin, isopropyl myristate, ethyl alcohol, and Tween 80 was prepared via spontaneous emulsification to increase the permeability and solubility of lutein. Physically transparent NEs with a particle size of ~10–12 nm with a narrow size distribution were selected as optimized formulations and neither particle size alteration nor phase separation was observed in these formulations for one week. The lutein-loaded NEs displayed a significant improvement in achieving the sustained release of lutein. It was stated in the study that the lutein-loaded NE formulation was capable of increasing the solubility, ocular retention, and permeability of lutein and showed promise as an ocular delivery system [150]. In another study, for the treatment of AMD, a lutein-loaded NE formulation was prepared and thereafter modified with stearyl-penetratin (NE-P) to achieve enhanced penetration to the posterior segment of the ocular region and improved distribution in the retina. Moreover, the nanosystem was reformulated as an ion-responsive in situ gelling system (NE-P GEL) in order to further increase the retention time on the cornea and analyzed with the fluorescence method to determine its length of retention in the eye. When compared to NE-P, the gel formulation displayed an immediate gelling tendency on the surface of the corneas of rats and facilitated drug delivery to the inner segments of the eye via prolonging the residence time of lutein in the ocular structure. Moreover, the effect of stearyl penetratin on permeability was evaluated through cellular uptake studies and intraocular distribution experiments. A rodent dry AMD model was used in one study to mimic the histopathology of retinal degeneration. The therapeutic efficacy was assessed by electroretinography (ERG) as well as by the level of ROS and the number of apoptotic cells in the eye. The results showed that NE-P was highly uptaken as compared to NE without penetratin, while the ROS levels and the apoptosis rate of the cells in the retina were significantly decreased. In efficacy experiments, the structure of the retina was also shown to be significantly improved without any toxicity; thus, it was revealed that a lutein-loaded NE-P in situ gelling system was a promising and non-invasive route for the treatment of AMD [151].

### 3.3. Solid Lipid Nanoparticles and Nanostructured Lipid Carriers

Solid lipid nanoparticles (SLNs) are nanosized colloidal carriers composed of lipids that are solid at room temperature and stabilized by emulsifiers in their aqueous phase [152]. Nanostructured lipid carriers (NLCs), considered to be the second generation of lipid carriers, combine liquid and solid lipids in their lipid matrix [153]. The combination of lipids lowers the melting point of the mixture because more disordered solid matrices are formed; this increases the loading capacity, minimizes the drug leakage during storage, and also leads to the drug being released in a more controlled manner. SLNs and NLCs may be produced reproducibly without toxic organic solvents using high-pressure homogenization or high-speed mixing techniques [154]. When compared to other particulate systems, they also have a number of advantages, including the biocompatibility and biodegradability of the materials, their low potential for toxicity, their improved physical stability, the ease of large-scale production, their ability to increase drug solubility, and their better control of drug release.

In a relatively early study, owing to its inadequate solubility, lutein was incorporated into lipid carriers for the purpose of dermal delivery. Lutein-loaded SLN, NLC, and nanoemulsion formulations were prepared using a high-pressure homogenization method. The mean size of the particles was between 150 and 350 nm, and this was reduced as the carriers’ lipid concentration increased. The smaller particle size of NLCs produced with higher liquid lipid levels was attributed to a reduction in the viscosity of the lipid matrix. The lipid NPs were found to be effective in protecting lutein against UV degradation. It was reported in this study that, after UV irradiation, only 0.06% lutein degradation was observed in SLN formulations and 6–8% in NLC ones, compared to the 50% lutein degradation found for powder dispersed in corn oil. The ability of SLNs to augment the stability of lutein, which is mainly localized in the particles’ core, was assumed to be responsible for this protective effect. Permeation trials conducted using fresh pig ear skin demonstrated that lutein was effectively kept in the skin and was not absorbed systemically, as would be expected from an ideal dermal formulation. Considering the size, release, and permeability results obtained as well as the chemical preservation of lutein before its absorption into the skin, researchers stated that, for lutein delivery, lipid NPs are promising dermal nano-vehicles [155].

In order to investigate the topical application of lutein for ocular delivery, Shah et al. successfully prepared lutein-loaded SLNs via hot homogenization and cold dilution techniques by using Gelucire^®^ 44/14. The improved formulation was optimized with an average particle size of 79.70 nm, and was able to maintain the drug release for 8 h in a medium imitating tear fluid. It was stated that, because lutein was highly soluble in Gelucire^®^ 44/14, an increase in the amount of this lipid in the formulation resulted in increased lutein entrapment. Additionally, as the amount of Gelucire^®^ increased, the mean particle size decreased, thereby increasing the effective surface area and the drug release rate. Ex vivo investigation revealed that the corneal hydration was 78.35%; hence, it was concluded that the optimized formulation had not caused any damage to the cornea [156]. In another research study by Tan et al. aiming to improve the stability and corneal permeability, lutein-loaded SLN formulations were developed utilizing an ultrasonic-assisted emulsion evaporation-low temperature curing technique and optimized via response surface methodology (RSM). In an optimized formulation prepared using glyceryl monostearate, lecithin, and poloxamer-188, the encapsulation efficiency was found to be 94.43%, and the average particle size was determined to be 118.50 nm. In particularly, the stability of the lutein loaded in SLNs against oxygen, light, and heat was enhanced by 3.21 times, 3.41 times, and 4.42 times, respectively, in comparison with free lutein. Release studies showed that lutein was released from SLNs in a sustained manner, and permeation studies revealed that lutein SLNs had an apparent permeability coefficient (Papp) 1.52 times higher than that of free lutein. It was noted in the study that, as an effective ophthalmic drug delivery system for lutein, SLNs could enhance the trans-corneal flux and ameliorate the stability of lutein [157].

Lacatusa et al. investigated the contribution of fish oil enriched with omega-3 fatty acids to producing stabilized NLCs with improved properties as efficient oral delivery systems for lutein. The mean particle size of the prepared NLC formulations was below 200 nm, and their entrapment efficiency was found to be 88.5%. According to the study, lutein-loaded NLCs made an important contribution to in vitro antioxidant activity, scavenging up to 98% of ROS. In this comparative research, NLC formulations outperformed traditional nanoemulsion formulations in terms of their in vitro sustained lutein release [158].

In another study focusing on the evaluation of the ocular delivery efficacy of lutein, the effects of NLCs and cyclodextrin (CD)-combined NLCs on corneal lutein accumulation were investigated by quantifying partition coefficients in the porcine cornea. CD-combined lipid NPs had drug loading efficiencies of up to 68% and also showed lower levels of cytotoxicity in bovine cornea cells. Furthermore, 2% CD-combined NLCs enhanced lutein accumulation up to a level of 209.2 ± 18 (μg/g), which was 4.9-fold higher than that of lipid NPs. It was stated in this study that the enhanced accumulation of lutein in the cornea was due to the permeation enhancement and nano-scaled properties of HP*β*CD-combined NLCs. Additionally, the CD-combined NLCs enhanced the stability and entrapment efficacy of lutein. Moreover, by raising the lutein payload in lipid NPs, the corneal lutein accumulation was also improved [159].

### 3.4. Polymer-Based Nanoparticles

Polymeric NPs are sub-micron colloidal particles that contain bioactive ingredients encapsulated in or conjugated to synthetic or natural polymeric macromolecular structures [160]. The bioavailability of active ingredients, especially lipophilic ones, with poor biopharmaceutical properties is known to be considerably improved by polymeric nanoparticles. Moreover, polymer-based nanocarriers possess advantages such as improved biocompatibility, enhanced stability, sustained, release and improved efficacy [161,162].

In investigations on polymeric NPs, the use of chitosan as a cationic natural biopolymer has received considerable attention in the food and pharmaceutical areas owing to its special qualities, including excellent biocompatibility, biodegradability, and safety [163]. Additionally, chitosan is preferably used in oral drug delivery systems alone or in combination with other polymers because it has the potential to increase absorption through the intestinal epithelial cell membrane due to its mucoadhesive and permeability-improving characteristics [164,165]. Regarding lutein-loaded chitosan NPs, in one study, water-soluble chitosan and poly-γ-glutamic acid-based NPs with a size of around 200 nm were shown to significantly enhance the solubility of encapsulated lutein as compared to the non-encapsulated form by a considerable amount [166]. In another study, lutein was entrapped in NPs developed by complexation between positively charged chitosan and negatively charged dextran sulphate in order to improve the sustained release to the ocular surface. In chitosan-dextran sulfate NPs with a mean size of ~400 nm, lutein’s entrapment efficiency was shown to be between 60% and 76%. These mucoadhesive NPs were demonstrated to increase the chemical stability and improve the ocular delivery of lutein [167]. On the other hand, in an in vivo study, lutein was encapsulated in water-soluble low-molecular-weight chitosan (LMWC) NPs. The oral bioavailability of this formulation, which was intended to be increased, was investigated in comparison with that of lutein-loaded mixed micelles. It was stated that micellar lutein was used as the control group in both in vitro and in vivo tests because lutein is incorporated into mixed micelles during the digestive process before being absorbed. The particle size of the nanocapsules ranged between 80 and 600 nm and lutein in the LMWC nanocapsules had a significantly higher (27.7%) bioavailability than the control. The particle size of NPs ranged between 80 and 600 nm and lutein in the LMWC NPs displayed a considerably higher (27.7%) bioavailability than micellar lutein. Moreover, the postprandial lutein levels in the liver (53.9%), plasma (54.5%), and eyes (62.8%) of mice fed nanoencapsulated lutein were found to be better than that of the control group. It was concluded that LMWC showed good potential as a delivery vehicle for improving the bioavailability of lutein in food and pharmaceutical applications [168].

Among diverse synthetic biodegradable polymers, poly(lactic-co-glycolic-acid) (PLGA) has been broadly studied due to its biocompatibility, stability in the digestive tract, and ability to provide sustained drug release [169,170]. In order to evaluate the ability of PLGA NPs to enhance lutein delivery, their bioavailability was assessed using an in vivo rat model. It is indicated in this study that, compared to free and micellized lutein, PLGA NPs improve the pharmacokinetics (Cmax and AUC) of lutein in the plasma and promote lutein accumulation in the mesenteric adipose tissue and spleen. Lutein uptake and release were also studied in intestinal Caco-2 cells; however, contrasting results were found compared to the in vivo study. Thus, it was suggested that the bioavailability of nanodelivered lutein could be improved more when combined with micelle components or fats in addition to PLGA NPs [171]. Other researchers have prepared PLGA nanoparticle formulations combined with polyethylene glycol (PEG) in order to improve the solubility, bioavailability, and antiproliferative properties of lutein. Their results suggested that the average particle size was about 200 nm and that the smaller size of nanocapsules would result in an overall larger surface area, which, in turn, would help to improve the aqueous solubility, intestinal permeability, and bioavailability of lutein. In an in vivo study, the postprandial plasma kinetics of an oral dose of lutein from nanocapsules was found to be higher compared to that of micellized lutein (control). Additionally, the anticancer activity of lutein from nanocapsules was investigated in human hepatocyte carcinoma (Hep G2) cells, and its antiproliferative effect was found to be higher than that of free lutein. Furthermore, the potential of PLGA-PEG nanocarriers to improve the bioavailability and antiproliferative properties of lutein was demonstrated in this study [172].

Besides the NPs summarized above, new-generation lutein-loaded NPs decorated with targeting agents have been introduced in the literature in recent years. In targeting strategies, the agent, which is predicted to be identified by the transporter proteins on the target cell membrane, is conjugated to a nanoparticle. The targeting-agent-decorated nanoparticles are then translocated at a higher level across the cell membrane, improving the drug’s permeability. Because the retinal epithelium possesses the transport system that uptakes biotin selectively, biotin-conjugated lutein-loaded PLGA-PEG NPs were prepared in order to increase the amount of lutein taken up by retinal cells. The successful encapsulation of lutein into PLGA and PLGA–PEG–biotin NPs with a homogeneous size distribution, below 250 nm, was achieved. The lutein entrapment efficiency was nearly 75% for PLGA–PEG–biotin NPs, which was higher than that of PLGA NPs (56%). It was confirmed in cellular uptake studies on human retinal pigment epithelial (ARPE-19) cells that a higher uptake of lutein could be achieved with PLGA–PEG–biotin NPs in comparison with PLGA NPs and lutein alone. Thus, it was suggested that PLGA–PEG–biotin polymeric NPs could be used as a potential delivery system for the treatment of AMD in order to achieve a higher uptake of lutein in retinal cells [173]. Furthermore, the same research group prepared lutein-loaded folate-decorated PLGA-PEG NPs and evaluated their uptake in the SK-N-BE(2) neuroblastoma cell line. As is widely known, the brain and blood–brain barrier express folate receptors, which can be used to actively target medications for neurological diseases such as brain cancer and hypoxic–ischemic encephalopathy (HIE). According to the results of this study, the average size of the stable lutein-loaded PLGA–PEG–FOLATE NPs was 188.0 nm and their lutein entrapment efficiency was 73%. It was shown in cellular uptake studies that lutein-loaded NPs were taken up by cells 1.6 and 2 times more than lutein alone and PLGA NPs, respectively. Additionally, it was confirmed that lutein-loaded PLGA–PEG–FOLATE NPs were taken up via folate-receptor-mediated endocytosis into neuroblastoma cells. Thus, it was noted that PLGA–PEG–FOLATE NPs could represent a novel therapeutic strategy for the delivery of lutein into brain tissues [174].

On the other hand, effective studies have been carried out on functionalizing lutein-loaded PLGA NPs by coating them with other biopolymers. For the treatment of AMD, in order to improve lutein delivery to ocular tissues by the intravitreal route of administration, hyaluronic acid (HA)-coated PLGA NPs were fabricated via a solvent displacement method. Suitably sized NPs with increased entrapment efficiency due to HA coating showed a biphasic release profile with an initial burst release of lutein followed by a constant rate of release. Since retinal pigment epithelial cells (ARPE-19) express CD44, which is a major HA receptor on the cell surface, the surface of lutein NPs was coated with HA to enhance the cellular binding of lutein NPs to CD44 on ARPE-19 cells. Additionally, HA provided a negative charge, hydrophilicity, and steric hindrance for stabilizing lutein NPs, as desired. Stability studies carried out at temperatures of 4 °C, 30 °C, and 40 °C revealed that lutein NPs should be stored at 4 °C to prevent physicochemical degradation. It was concluded in this study that HA-coated PLGA NPs can be used as an ocular lutein delivery system to provide sustained release and prolong the availability of lutein in the vitreous humor by efficiently binding to ARPE-19 cells [175]. In a recent comprehensive study, it was reported that the delivery of lutein NPs to the brain could be realized through the nasal route. PLGA NPs were designed for the intranasal delivery of lutein using the nanoprecipitation method and by coating them with chitosan in order to reduce oxidative stress in patients with Alzheimer’s Disease (AD). The spherical NPs, which were uniformly coated with cationic chitosan via electrostatic interaction over the PLGA core, displayed a mean particle size of below 150 nm and a lutein entrapment efficiency of more than 80%. The optimized NPs were extensively investigated by in vitro cytotoxicity, cellular uptake, as well as blood–brain–barrier (BBB) permeation studies. By conjugating fluorescein isothiocyanate (FITC) to the surface of NPs, the in vivo biodistribution of NPs was investigated and their excellent permeation capacity through the nasal mucosal membrane was discovered. It was revealed in cellular uptake studies that caveolae-mediated endocytosis increased the internalization of NPs. Moreover, it was shown in a co-culture model of BBB that the efficient passage of NPs was obtained across the BBB. The considerable ROS scavenging ability of NPs was shown using an antioxidant assay, while the safety of NPs was confirmed by an in vivo toxicity study. Furthermore, NPs were found to be highly deposited in the brain in a study of their in vivo bio-distribution after application via the intranasal route. Therefore, lutein-loaded chitosan-coated PLGA NPs exhibited excellent potential to be used as nanocarrier systems that could be targeted to the brain for the treatment of AD [176].

### 3.5. Polymer/Lipid-Based Nanoparticles

Researchers have suggested that understanding the mechanisms of intestinal uptake of lutein is critical in order to improve the efficiency of lutein carriers [177]. It is worth repeating that, due to its lipophilic nature, lutein is dispersed in dietary oils and included in mixed micelles for intestinal absorption, as the oil drops work as a delivery vehicle and facilitate the solubilization of lutein, making it accessible during digestion [178]. Polymer- and lipid-based delivery systems are two prevalent drug carrier systems with their own advantages and disadvantages. Regarding lutein delivery, the polymer ensures the stability of the encapsulated lutein by protecting it against extreme pH conditions and other dietary elements that can interfere with lutein absorption. On the other hand, the lipid moiety, acting as an emulsifier, improves the solubility, encapsulation efficiency, and bioavailability of lutein [179]. Based on this approach, polymer–lipid hybrid systems have received considerable attention as state-of-art nanocarriers for lutein delivery [180].

In this regard, in order to obtain hybrid system benefits, phospholecithin (PL), as a phospholipid, was included in a lutein–PLGA nanoformulation. The average particle size of PLGA–PL NPs was approximately 140 nm, and the in vitro lutein release exhibited an initial burst followed by sustained lutein release up to 86%. It was observed that the in vitro bioaccessibility of nano-entrapped lutein was 62.7% higher than that of free lutein. In an in vivo oral pharmacokinetic study conducted in mice, the AUC of lutein after the application of a single oral dose of lutein–PLGA–PL NPs showed 3.91-fold (plasma), 2.89-fold (liver), and 3.12-fold (eyes) higher levels of absorption than micellized lutein, which was used as a control group. Additionally, the cellular uptake and anti-proliferative activity were studied in Hep G2 cells, where the lutein–PLGA–PL NPs displayed more effective anti-tumor efficacy. The higher cellular uptake of lutein from PLGA–PL NPs was attributed to the presence of PL in the formulation, as this helps in improving the absorption of lutein. The higher anti-proliferative activity found was attributed to the higher lutein uptake, prolonged activity, and stability of lutein within the nanoparticles. It was also proposed in this study that the inclusion of PL in the carrier system provided an advantage even in the presence of health complications causing an inadequate secretion of bile. It was concluded by the researchers that PLGA–PL hybrid nanoparticles could be used as an efficient delivery system, combining the advantages of polymer and lipid nanocarriers to achieve the improved solubility, stability, bioavailability, and anti-proliferative activity of lutein [181].

Natural polymers have also been investigated with regard to the preparation of polymer–lipid hybrid systems in order to improve the solubility, stability, and bioavailability of lutein. In one study, chitosan–sodium alginate-based NPs were fabricated via the ionotropic gelation method, and oleic acid was incorporated into the system. Lutein-loaded chitosan–sodium alginate–oleic acid hybrid NPs had sizes within the range of 40–160 nm together with the desired zeta potential. The hybrid NPs ensured a 1000-fold higher aqueous solubility together with an in vitro sustained release of lutein. Moreover, the hybrid NPs displayed negligible cytotoxicity against Caco-2 cells and a significantly higher intracellular transport (40%) of lutein into the Caco-2 monolayers. In the meantime, lutein from hybrid NPs exhibited a 128.3% improved oral bioavailability compared to micellar lutein in an in vivo pharmacokinetic study conducted on rats using a single dose via oral gavage. The excellent results achieved were attributed to the nano-size, positive surface charge, and lipid moiety of hybrid NPs, which contributed to the stability, solubility, and intestinal permeability and bioavailability of lutein. Thus, it was suggested that a chitosan–sodium alginate–oleic acid delivery system could be an efficient therapeutic tool to ameliorate the effects of degenerative diseases such as AMD and retinopathy [182].

In another study, Shwetha et al. focused on improving the oral delivery of lutein and prepared lutein-loaded chitosan hybrid NPs with the inclusion of phosphatidylcholine (PC). With regard to hybrid NPs, the contribution of PC comprising two long-chain acyl moieties and an abundance of oleic acid was clearly stated. At acidic pH, the presence of PC reduced the release of lutein, whereas at basic pH in the intestine, PC-coated NPs displayed higher levels of lutein release and cellular uptake in Caco-2 cells compared to uncoated NPs and micellar lutein. The inclusion of PC in NPs helped to preserve the lutein in an amorphous state, enhancing the lutein solubility as well as its bioavailability. Moreover, PC helped with the stabilization of NPs by increasing the surface charge and preventing particle agglomeration. Furthermore, acting as a surfactant, PC reduced the particle size and increased the encapsulation efficiency. It was claimed in this study that lutein-encapsulated chitosan–PC hybrid NPs significantly improved the bioavailability of lutein at enterocyte levels and showed promise for use in the prevention of AMD and other degenerative diseases [183]. Table 1 represents a comparison of nanoscale delivery systems on the bioavailability of lutein in rodent models.

## 4. Conclusions

Lutein is known to accumulate preferentially in the macular region of the human retina and protect the macula from light-initiated oxidative damage. Lutein contains an extended conjugated double-bond system together with hydroxyl groups at each end of the chromophore, which are responsible for its excellent light-absorbing properties. The anti-oxidant and anti-inflammatory effects of lutein are attributed to its unique structure, which is thought to be responsible for its ability to inhibit the development of not only ocular diseases such as AMD and cataracts, but also degenerative disorders such as cancer, atherosclerosis, and neurodegenerative diseases. Until recently, lutein has mainly been investigated in the context of eye-related diseases. Today, it draws considerable interest with regard to the prevention of age-related and degenerative disorders, and its therapeutic potential will soon move well beyond the realm of eye health. As we learn more about lutein’s cytoprotective and disease prevention activities, the interest in its therapeutic efficacy will increase. In addition, at present there is expanding interest in the design and production of nanoscale delivery systems for the encapsulation of natural bioactive compounds to enhance their stability and bioavailability. For the delivery of lutein, the potential of nanotechnology-based approaches should be evaluated in comprehensive clinical studies. Recent developments in the field of nanoscale delivery systems have revealed their promising outcomes. The fact remains that the success of these systems depends on their safety, cost, and bench-to-bedside transfer. The increasing success of nanoscale delivery systems will pave the way for the improvement of lutein’s therapeutic efficacy and its clinical translation.

## Figures and Tables

**Table 1:** Effect of nanoscale delivery systems on the bioavailability of lutein in rodent models.

Nanoscale Delivery System	Carrier	Size (nm)	Encapsulation Efficiency (%)	In Vivo Results	Ref.
**Emulsion-based systems**					
	NE (medium-chain triglyceride,α-tocopheryl polyethylene glycol succinate)	254.2	NA	As compared to lutein powder, the nanoemulsion increased the level of lutein in the liver and plasma by 1.6- and 2-fold, respectively.	[147]
**Polymer-based NPs**					
	Chitosan (LMWC)	80–600	85 ± 1	NPs displayed a considerably higher (27.7%) bioavailability than micellar lutein. Moreover, the postprandial lutein level in the liver (53.9%), plasma (54.5%), and eyes (62.8%) of mice fed NPs were found better than that of the control	[168]
	PLGA	124 ± 4	52 ± 3	NPs improve the pharmacokinetics (Cmax and AUC) of lutein in the plasma and promote lutein accumulation in the mesenteric adipose tissue and spleen, compared to free and micellized lutein	[171]
	PLGA-PEG	80–500 (~200)	88 ± 2	The postprandial plasma kinetics of an oral dose of lutein from NPs was found to be higher compared with that of micellized lutein	[172]
**Polymer/Lipid-Based NPs**					
	PLGA-PL	140 ± 6	90 ± 2	AUC of lutein after the application of a NPs showed 3.91-fold (plasma), 2.89-fold (liver), and 3.12-fold (eyes) higher levels of absorption than micellized lutein	[181]
	CHI-OL-ALG	40–160 (~200)	NA	In the oral pharmacokinetic study, using a single dose via oral gavage, lutein from NPs exhibited a 128.3% improved oral bioavailability compared to micellar lutein	[182]

NP: Nanoparticle; NE: nanoemulsion; LMWC: low-molecular-weight chitosan; PLGA: polylactic-co-glycolic acid; PEG: polyethylene glycol; PL: phospholipid; CHI-OA-ALG: chitosan-oleic acid-sodium alginate; PVP: polyvinylpyrrolidone; NA: Not available. Cmax: maximum concentration; AUC: area under the concentration-time curve.

## Data Availability

Not applicable.

## References

[1] Moosavi M.A., Haghi A., Rahmati M., Taniguchi H., Mocan A., Echeverria J., Gupta V.K., Tzvetkov N.T., Atanasov A.G. (2018). Phytochemicals as potent modulators of autophagy for cancer therapy. Cancer Lett..

[2] Liu R.H. (2004). Potential synergy of phytochemicals in cancer prevention: Mechanism of action. J. Nutr..

[3] Saini R.K., Prasad P., Lokesh V., Shang X., Shin J., Keum Y.-S., Lee J.-H. (2022). Carotenoids: Dietary sources, extraction, encapsulation, bioavailability, and health benefits—A review of recent advancements. Antioxidants.

[4] Tan B.L., Norhaizan M.E. (2019). Carotenoids: How effective are they to prevent age-related diseases?. Molecules.

[5] Maiani G., Caston M.J., Catasta G., Toti E., Cambrodon I.G., Bysted A., Granado-Lorencio F., Olmedilla-Alonso B., Knuthsen P., Valoti M. (2009). Carotenoids: Actual knowledge on food sources, intakes, stability and bioavailability and their protective role in humans. Mol. Nutr. Food Res..

[6] Namitha K.K., Negi P.S. (2010). Chemistry and biotechnology of carotenoids. Crit. Rev. Food Sci. Nutr..

[7] Landrum J.T., Bone R.A. (2001). Lutein, zeaxanthin, and the macular pigment. Arch. Biochem. Biophys..

[8] Cazzaniga S., Bressan M., Carbonera D., Agostini A., Dall’Osto L. (2016). Diferential roles of carotenes and xanthophylls in photosystem I photoprotection. Biochemistry.

[9] Alves-Rodrigues A., Shao A. (2004). The science behind lutein. Toxicol. Lett..

[10] Park H.-A., Hayden M.M., Bannerman S., Jansen J., Crowe-White K.M. (2020). Anti-apoptotic effects of carotenoids in neurodegeneration. Molecules.

[11] Snodderly D.M., Auran J.D., Delori F.C. (1984). The macular pigment. II. Spatial distribution in primate retinas. Investig. Ophthalmol. Vis. Sci..

[12] Arunkumar R., Calvo C.M., Conrady C.D., Bernstein P.S. (2018). What do we know about the macular pigment in AMD: The past, the present, and the future. Eye.

[13] Johra F.T., Bepari A.K., Bristy A.T., Reza H.M. (2020). A mechanistic review of β-carotene, lutein, and zeaxanthin in eye health and disease. Antioxidants.

[14] Al Mijan M., Sim W.-J., Lim T.-G. (2021). Physiological Effects of Green-Colored Food-Derived Bioactive Compounds on Cancer. Appl. Sci..

[15] Bernstein P.S., Li B., Vachali P.P., Gorusupudi A., Shyam R., Henriksen B.S., Nolan J.M. (2016). Lutein, zeaxanthin, and meso-zeaxanthin: The basic and clinical science underlying carotenoid-based nutritional interventions against ocular disease. Prog. Retin. Eye Res..

[16] Abdel-Aal E.-S.M., Akhtar H., Zaheer K., Ali R. (2013). Dietary sources of lutein and zeaxanthin carotenoids and their role in eye health. Nutrients.

[17] Roberts R.L., Green J., Lewis B. (2009). Lutein and zeaxanthin in eye and skin health. Clin. Dermatol..

[18] Demmig-Adams B., López-Pozo M., Stewart J.J., Adams W.W. (2020). Zeaxanthin and lutein: Photoprotectors, anti-inflammatories, and brain food. Molecules.

[19] Hammond B.R., Wooten B.R., Snodderly D.M. (1997). Individual variations in the spatial profile of human macular pigment. J. Opt. Soc. Am. B.

[20] Subczynski W., Wisniewska A., Widomska J. (2010). Location of macular xanthophylls in the most vulnerable regions of photoreceptor outer-segment membranes. Arch. Biochem. Biophys..

[21] Lem D.W., Davey P.G., Gierhart D.L., Rosen R.B. (2021). A systematic review of carotenoids in the management of age-related macular degeneration. Antioxidants.

[22] Mrowicka M., Mrowicki J., Kucharska E., Majsterek I. (2022). Lutein and zeaxanthin and their roles in age-related macular degeneration—Neurodegenerative disease. Nutrients.

[23] Lem D.W., Gierhart D.L., Davey P.G. (2021). A systematic review of carotenoids in the management of diabetic retinopathy. Nutrients.

[24] Manayi A., Abdollahi M., Raman T., Nabavi S.F., Habtemariam S., Daglia M., Nabavi S.M. (2016). Lutein and cataract: From bench to bedside. Crit. Rev. Biotechnol..

[25] Shegokar R., Mitri K. (2012). Carotenoid lutein: A promising candidate for pharmaceutical and nutraceutical applications. J. Diet. Suppl..

[26] Madaan T., Choudhary A.N., Gyenwalee S., Thomas S., Mishra H., Tariq M., Vohora D., Talegaonkar S. (2017). Lutein, a versatile phyto-nutraceutical: An insight on pharmacology, therapeutic indications, challenges and recent advances in drug delivery. Pharma Nutr..

[27] De Felice S.L. (1995). The nutraceutical revolution: Its impact on food industry R&D. Trends Food Sci. Technol..

[28] Santini A., Novellino E. (2017). To Nutraceuticals and Back: Rethinking a Concept. Foods.

[29] Santini A., Novellino E. (2014). Nutraceuticals: Beyond the Diet before the Drugs. Curr. Bioact. Compd..

[30] Santini A., Tenore G.C., Novellino E. (2017). Nutraceuticals: A paradigm of proactive medicine. Eur. J. Pharm. Sci..

[31] Santini A., Novellino E. (2017). Nutraceuticals in hypercholesterolaemia: An Overview. Br. J. Pharmacol..

[32] Daliu P., Santini A., Novellino E. (2019). From pharmaceuticals to nutraceuticals: Bridging disease prevention and management. Expert Rev. Clin. Pharmacol..

[33] Santini A., Cammarata S.M., Capone G., Ianaro A., Tenore G.C., Pani L., Novellino E. (2018). Nutraceuticals: Opening the debate for a regulatory framework. Br. J. Clin. Pharmacol..

[34] Santini A., Novellino E. (2018). Nutraceuticals: Shedding light on the grey area between pharmaceuticals and food. Expert Rev. Clin. Pharmacol..

[35] Arunkumar R., Gorusupudi A., Bernstein P.S. (2020). The macular carotenoids: A biochemical overview. Biochim. Biophys. Acta Mol. Cell Biol. Lipids.

[36] Mitra S., Rauf A., Tareq A.M., Jahan S., Emran T.B., Shahriar T.G., Dhama K., Alhumaydhi F.A., Aljohani A.S.M., Rebezov M. (2021). Potential health benefits of carotenoid lutein: An updated review. Food Chem. Toxicol..

[37] Li L.H., Lee J.C., Leung H.H., Lam W.C., Fu Z., Lo A.C.Y. (2020). Lutein supplementation for eye diseases. Nutrients.

[38] Wong K.-H., Nam H.-Y., Lew S.-Y., Naidu M., David P., Kamalden T.A., Hadie S.N.H., Lim L.-W. (2022). Discovering the Potential of Natural Antioxidants in Age-Related Macular Degeneration: A Review. Pharmaceuticals.

[39] McClements D.J. (2015). Nanoscale nutrient delivery systems for food applications: Improving bioactive dispersibility, stability, and bioavailability. J. Food Sci..

[40] Zhang Y., Kong L., Tan L. (2020). Effectiveness of nanoscale delivery systems on improving the bioavailability of lutein in rodent models: A systematic review. Crit. Rev. Food Sci. Nutr..

[41] Conn P.F., Schalch W., Truscott T.G. (1991). The singlet oxygen and carotenoid interaction. J. Photochem. Photobiol. B.

[42] Demmig-Adams B., Polutchko S.K., Adams W.W. (2022). Structure-function-environment relationship of the isomers zeaxanthin and lutein. Photochem.

[43] Becerra M.O., Contreras L.M., Lo M.H., Diaz J.M., Herrera G.C. (2020). Lutein as a functional food ingredient: Stability and bioavailability. J. Funct. Foods.

[44] Widomska J., Zareba M., Subczynski W.K. (2016). Can xanthophyll-membrane interactions explain their selective presence in the retina and brain?. Foods.

[45] Rodriguez-Amaya D.B. (2016). Natural food pigments and colorants. Curr. Opin. Food Sci..

[46] Ahn Y.J., Kim H. (2021). Lutein as a modulator of oxidative stress-mediated inflammatory diseases. Antioxidants.

[47] Kijlstra A., Tian Y., Kelly E.R., Berendschot T.T. (2012). Lutein: More than just a filter for blue light. Prog. Retin. Eye Res..

[48] Sujak A., Gabrielska J., Grudzinski W., Borc R., Mazurek P., Gruszecki W.I. (1999). Lutein and zeaxanthin as protectors of lipid membranes against oxidative damage: The structural aspects. Arch. Biochem. Biophys..

[49] Gabrielska J., Gruszecki W.I. (1996). Zeaxanthin (dihydroxy-β-carotene) but not β-carotene rigidifies lipid membranes: A ^1^H-NMR study of carotenoid-egg phosphatidylcholine liposomes. Biochim. Biophys. Acta.

[50] Gruszecki W.I., Strzałka K. (2005). Carotenoids as modulators of lipid membrane physical properties. Biochim. Biophys. Acta.

[51] Grudzinski W., Nierzwicki L., Welc R., Reszczynska E., Luchowski R., Czub J., Gruszecki W.I. (2017). Localization and orientation of xanthophylls in a lipid bilayer. Sci. Rep..

[52] Widomska J., Subczynski W.K. (2014). Why has nature chosen lutein and zeaxanthin to protect the retina?. J. Clin. Exp. Ophthalmol..

[53] Ranard K.M., Jeon S., Mohn E.S., Griffiths J.C., Johnson E.J., Erdman J.W. (2017). Dietary guidance for lutein: Consideration for intake recommendations is scientifically supported. Eur. J. Nutr..

[54] Sauer L., Li B., Bernstein P.S. (2019). Ocular carotenoid status in health and disease. Annu. Rev. Nutr..

[55] Sommerburg O., Keunen J.E.E., Bird A.C., Van Kuijk F.J.G.M. (1998). Fruits and vegetables that are sources for lutein and zeaxanthin: The macular pigment in human eyes. Br. J. Ophthalmol..

[56] During A., Doraiswamy S., Harrison E.H. (2008). Xanthophylls are preferentially taken up compared with β-carotene by retinal cells via a SRBI-dependent mechanism. J. Lipid Res..

[57] Yoo J.H., Shanmugam S., Thapa P., Lee E.S., Balakrishnan P., Baskaran R., Yoon S.K., Choi H.G., Yong C.S., Yoo B.K. (2010). Novel self-nanoemulsifying drug delivery system for enhanced solubility and dissolution of lutein. Arch. Pharm. Res..

[58] Buscemi S., Corleo D., Di Pace F., Petroni M.L., Satriano A., Marchesini G. (2018). The effect of lutein on eye and extra-eye health. Nutrients.

[59] Weigert G., Kaya S., Pemp B., Sacu S., Lasta M., Werkmeister R.M., Dragostinoff N., Simader C., Garhöfer G., Schmidt-Erfurth U. (2011). Effects of lutein supplementation on macular pigment optical density and visual acuity in patients with age-related macular degeneration. Investig. Ophthalmol. Vis. Sci..

[60] Koushan K., Rusovici R., Li W., Ferguson L.R., Chalam K.V. (2013). The role of lutein in eye-related disease. Nutrients.

[61] Richer S., Stiles W., Statkute L., Pulido J., Frankowski J., Rudy D., Pei K., Tsipursky M., Nyland J. (2004). Double-masked placebo controlled randomized trial of lutein and antioxidant supplementation in the intervention of atrophic age-related macular degeneration the Veterans LAST study (Lutein Antioxidant Supplementation Trial). Optometry.

[62] Bian Q., Qin T., Ren Z., Wu D., Shang F. (2012). Lutein or zeaxanthin supplementation suppresses inflammatory responses in retinal pigment epithelial cells and macrophages. Adv. Exp. Med. Biol..

[63] Kamoshita M., Toda E., Osada H., Narimatsu T., Kobayashi S., Tsubota K., Ozawa Y. (2016). Lutein acts via multiple antioxidant pathways in the photo-stressed retina. Sci. Rep..

[64] Liu H., Liu W., Zhou X., Long C., Kuang X., Hu J., Tang Y., Liu L., He J., Huang Z. (2017). Protective effect of lutein on ARPE-19 cells upon H_2_O_2_-induced G2/M arrest. Mol. Med. Rep..

[65] Padmanabha S., Vallikannan B. (2018). Fatty acids modulate the efficacy of lutein in cataract prevention: Assessment of oxidative and inflammatory parameters in rats. Biochem. Biophys. Res. Commun..

[66] Krinsky N.I., Landrum J.I., Bone R.A. (2003). Biological mechanisms of the protective role of lutein and zeaxanthin in the eye. Ann. Rev. Nutr..

[67] Fuad N.I.N., Sekar M., Gan S.H., Lum P.T., Vaijanathappa J., Ravi S. (2020). Lutein: A comprehensive review on its chemical, biological activities and therapeutic potentials. Pharmacogn. J..

[68] Park J.S., Chew B.P., Wong T.S. (1998). Dietary lutein from marigold extract inhibits mammary tumor development in BALB/c mice. J. Nutr..

[69] Chew B.P., Brown C.M., Park J.S., Mixter P.F. (2003). Dietary lutein inhibits mouse mammary tumor growth by regulating angiogenesis and apoptosis. Anticancer Res..

[70] Sindhu E.R., Firdous A.P., Ramnath V., Kuttan R. (2013). Effect of carotenoid lutein on *N*-nitrosodiethylamine-induced hepatocellular carcinoma and its mechanism of action. Eur. J. Cancer Prev..

[71] Gong X., Smith J., Swanson H., Rubin L. (2018). Carotenoid Lutein Selectively Inhibits Breast Cancer Cell Growth and Potentiates the Effect of Chemotherapeutic Agents through ROS-Mediated Mechanisms. Molecules.

[72] Rafi M.M., Kanakasabai S., Gokarn S.V., Krueger E.G., Bright J.J. (2015). Dietary lutein modulates growth and survival genes in prostate cancer cells. J. Med. Food.

[73] Li Y., Zhang Y., Liu X., Wang M., Wang P., Yang J., Zhang S. (2018). Lutein inhibits proliferation, invasion and migration of hypoxic breast cancer cells via downregulation of HES1. Int. J. Oncol..

[74] Zhang W.-L., Zhao Y.-N., Shi Z.-Z., Cong D., Bai Y.-S. (2018). Lutein inhibits cell growth and activates apoptosis via the PI3K/AKT/mTOR signaling pathway in A549 human non-small-cell lung cancer cells. J. Environ. Pathol. Toxicol. Oncol. Off. Organ. Int. Soc. Environ. Toxicol. Cancer.

[75] Gansukh E., Mya K.K., Jung M., Keum Y.S., Kim D.H., Saini R.K. (2019). Lutein derived from marigold (*Tagetes erecta*) petals triggers ROS generation and activates Bax and caspase-3 mediated apoptosis of human cervical carcinoma (HeLa) cells. Food Chem. Toxicol..

[76] Kavalappa Y.P., Gopal S.S., Ponesakki G. (2021). Lutein inhibits breast cancer cell growth by suppressing antioxidant and cell survival signals and induces apoptosis. J. Cell. Physiol..

[77] Shin J., Song M.-H., Oh J.-W., Keum Y.-S., Saini R.K. (2020). Pro-oxidant actions of carotenoids in triggering apoptosis of cancer cells: A review of emerging evidence. Antioxidants.

[78] Mecocci P., Polidori M.C., Cherubini A., Ingegni T., Mattioli P., Catani M., Rinaldi P., Cecchetti R., Stahl W., Senin U. (2002). Lymphocyte oxidative DNA damage and plasma antioxidants in Alzheimer disease. Arch. Neurol..

[79] Rinaldi P., Polidori M.C., Metastasio A., Mariani E., Mattioli P., Cherubini A., Catani M., Cecchetti R., Senin U., Mecocci P. (2003). Plasma antioxidants are similarly depleted in mild cognitive impairment and in Alzheimer’s disease. Neurobiol. Aging.

[80] Min J.Y., Min K.B. (2014). Serum lycopene, lutein and zeaxanthin, and the risk of Alzheimer’s disease mortality in older adults. Dement. Geriatr. Cogn. Disord..

[81] Feeney J., O’Leary N., Moran R., O’Halloran A.M., Nolan J.M., Beatty S., Young I.S., Kenny R.A. (2017). Plasma lutein and zeaxanthin are associated with better cognitive function across multiple domains in a large population-based sample of older adults: Findings from the Irish longitudinal study on aging. J. Gerontol. A.

[82] Tan D., Yu X., Chen M., Chen J., Xu J. (2017). Lutein protects against severe traumatic brain injury through anti-inflammation and antioxidative effects via ICAM-1/Nrf-2. Mol. Med. Rep..

[83] Wu W., Li Y., Wu Y., Zhang Y., Wang Z., Liu X. (2015). Lutein suppresses inflammatory responses through Nrf2 activation and NF-κB inactivation in lipopolysaccharide-stimulated BV-2 microglia. Mol. Nutr. Food Res..

[84] Shimazu Y., Kobayashi A., Endo S., Takemura J., Takeda M. (2019). Effect of lutein on the acute inflammation-induced c-Fos expression of rat trigeminal spinal nucleus caudalis and C1 dorsal horn neurons. Eur. J. Oral. Sci..

[85] Stringham J.M., Johnson E.J., Hammond B.R. (2019). Lutein across the lifespan: From childhood cognitive performance to the aging eye and brain. Curr. Dev. Nutr..

[86] Dwyer J.H., Navab M., Dwyer K.M., Hassan K., Sun P., Shircore A., Hama-Levy S., Hough G., Wang X., Drake T. (2001). Oxygenated carotenoid lutein and progression of early atherosclerosis: The Los Angeles atherosclerosis study. Circulation.

[87] Zou Z., Xu X., Huang Y., Xiao X., Ma L., Sun T., Dong P., Wang X., Lin X. (2011). High serum level of lutein may be protective against early atherosclerosis: The Beijing atherosclerosis study. Atherosclerosis.

[88] Chung R.W.S., Leanderson P., Lundberg A.K., Jonasson L. (2017). Lutein exerts anti-inflammatory effects in patients with coronary artery disease. Atherosclerosis.

[89] Lidebjer C., Leanderson P., Ernerudh J., Jonasson L. (2007). Low plasma levels of oxygenated carotenoids in patients with coronary artery disease. Nutr. Metab. Cardiovasc. Dis..

[90] Howard A.N., Thurnham D.I. (2017). Lutein and atherosclerosis: Belfast versus Toulouse revisited. Med. Hypotheses.

[91] Shanmugasundaram R., Selvaraj R.K. (2011). Dietary lutein and fish oil interact to alter atherosclerotic lesions in a Japanese quail model of atherosclerosis. J. Anim. Physiol. Anim. Nutr..

[92] Ford E.S., Gillespie C., Ballew C., Sowell A., Mannino D. (2002). Serum carotenoid concentrations in US children and adolescents. Am. J. Clin. Nutr..

[93] Gopal S.S., Eligar S.M., Vallikannan B., Ponesakki G. (2021). Inhibitory efficacy of lutein on adipogenesis is associated with blockage of early phase regulators of adipocyte differentiation. Biochim. Biophys. Acta Mol. Cell. Biol. Lipids.

[94] Ford E.S., Mokdad A.H., Giles W.H., Brown D.W. (2003). The metabolic syndrome and antioxidant concentrations: Findings from the Third National Health and Nutrition Examination Survey. Diabetes.

[95] Sluijs I., Beulens J.W., Grobbee D.E., van der Schouw Y.T. (2009). Dietary carotenoid intake is associated with lower prevalence of metabolic syndrome in middle-aged and elderly men. J. Nutr..

[96] Tuzcu M., Orhan C., Muz O.E., Sahin N., Juturu V., Sahin K. (2017). Lutein and zeaxanthin isomers modulates lipid metabolism and the inflammatory state of retina in obesity-induced high-fat diet rodent model. BMC Ophthalmol..

[97] Giordano E., Quadro L. (2018). Lutein, zeaxanthin and mammalian development: Metabolism, functions and implications for health. Arch. Biochem. Biophys..

[98] Ozawa Y., Sasaki M., Takahashi N., Kamoshita M., Miyake S., Tsubota K. (2012). Neuroprotective effects of lutein in the retina. Curr. Pharm. Des..

[99] Ribaya-Mercado J.D., Blumberg J.B. (2004). Lutein and zeaxanthin and their potential roles in disease prevention. J. Am. Coll. Nutr..

[100] Sasaki M., Ozawa Y., Kurihara T., Noda K., Imamura Y., Kobayashi S., Ishida S., Tsubota K. (2009). Neuroprotective effect of an antioxidant, lutein, during retinal inflammation. Investig. Ophthalmol. Vis. Sci..

[101] Ma L., Zhang M., Zhao R., Wang D., Ma Y., Ai L. (2021). Plant natural products: Promising resources for cancer chemoprevention. Molecules.

[102] Tanaka T., Shnimizu M., Moriwaki H. (2012). Cancer chemoprevention by carotenoids. Molecules.

[103] Kaulmann A., Bohn T. (2014). Carotenoids, inflammation, and oxidative stress—Implications of cellular signaling pathways and relation to chronic disease prevention. Nutr. Res..

[104] Furman D., Campisi J., Verdin E., Carrera-Bastos P., Targ S., Franceschi C., Ferrucci L., Gilroy D.W., Fasano A., Miller G.W. (2019). Chronic inflammation in the etiology of disease across the life span. Nat. Med..

[105] Jia Y.-P., Sun L., Yu H.-S., Liang L.-P., Li W., Ding H., Song X.-B., Zhang L.-J. (2017). The pharmacological effects of lutein and zeaxanthin on visual disorders and cognition diseases. Molecules.

[106] Liu T., Liu W.H., Zhao J.S., Meng F.Z., Wang H. (2017). Lutein protects against β-amyloid peptide-induced oxidative stress in cerebrovascular endothelial cells through modulation of Nrf-2 and NF-κb. Cell. Biol. Toxicol..

[107] Li S., Ding Y., Niu Q., Xu S., Pang L., Ma R., Jing M., Feng G., Tang J.X., Zhang Q. (2015). Lutein has a protective effect on hepatotoxicity induced by arsenic via Nrf2 signaling. BioMed Res. Int..

[108] Rafi M.M., Shafaie Y. (2007). Dietary lutein modulates inducible nitric oxide synthase (iNOS) gene and protein expression in mouse macrophage cells (RAW 264.7). Mol. Nutr. Food Res..

[109] Choi J.S., Kim D., Hong Y.M., Mizuno S., Joo C.K. (2006). Inhibition of nNOS and COX-2 expression by lutein in acute retinal ischemia. Nutrition.

[110] Du S.Y., Zhang Y.L., Bai R.X., Ai Z.L., Xie B.S., Yang H.Y. (2015). Lutein prevents alcohol-induced liver disease in rats by modulating oxidative stress and inflammation. Int. J. Clin. Exp. Med..

[111] Kim J.E., Clark R.M., Park Y., Lee J., Fernandez M.L. (2012). Lutein decreases oxidative stress and inflammation in liver and eyes ofguinea pigs fed a hypercholesterolemic diet. Nutr. Res. Pract..

[112] Muriach M., Bosch-Morell F., Arnal E., Alexander G., Blomhoff R., Romero F.J. (2008). Lutein prevents the effect of high glucose levels on immune system cells in vivo and in vitro. J. Physiol. Biochem..

[113] Kim J.-H., Na H.-J., Kim C.-K., Kim J.-Y., Ha K.-S., Lee H., Chung H.-T., Kwon H.J., Kwon Y.-G., Kim Y.-M. (2008). The non-provitamin A carotenoid, lutein, inhibits NF-κB-dependent gene expression through redox-based regulation of the phosphatidylinositol 3-kinase/PTEN/Akt and NF-κB-inducing kinase pathways: Role of H_2_O_2_ in NF-κB activation. Free Radic. Biol. Med..

[114] Chang J., Zhang Y., Li Y., Lu K., Shen Y., Guo Y., Qi Q., Wang M., Zhang S. (2018). NrF2/ARE and NF-κB pathway regulation may be the mechanism for lutein inhibition of human breast cancer cell. Future Oncol..

[115] Ouyang B., Li Z., Ji X., Huang J., Zhang H., Jiang C. (2019). The protective role of lutein on isoproterenol-induced cardiac failure rat model through improving cardiac morphology, antioxidant status via positively regulating Nrf2/HO-1 signalling pathway. Pharm. Biol..

[116] Qiao Y.Q., Jiang P.F., Gao Y.Z. (2018). Lutein prevents osteoarthritis through Nrf2 activation and downregulation of inflammation. Arch. Med. Sci..

[117] Zhao C., Shen X., Guo M. (2018). Stability of lutein encapsulated whey protein nano-emulsion during storage. PLoS ONE.

[118] Sridhar K., Inbaraj B.S., Chen B.-H. (2021). Recent Advances on Nanoparticle Based Strategies for Improving Carotenoid Stability and Biological Activity. Antioxidants.

[119] Souto E.B., Silva G.F., Dias-Ferreira J., Zielinska A., Ventura F., Durazzo A., Lucarini M., Novellino E., Santini A. (2020). Nanopharmaceutics: Part I—Clinical Trials Legislation and Good Manufacturing Practices (GMP) of Nanotherapeutics in the EU. Pharmaceutics.

[120] Durazzo A., Nazhand A., Lucarini M., Atanasov A.G., Souto E.B., Novellino E., Capasso R., Santini A. (2020). An Updated Overview on Nanonutraceuticals: Focus on Nanoprebiotics and Nanoprobiotics. Int. J. Mol. Sci..

[121] Yeung A.W.K., Souto E.B., Durazzo A., Lucarini M., Novellino E., Tewari D., Wang D., Atanasov A.G., Santini A. (2020). Big impact of nanoparticles: Analysis of the most cited nanopharmaceuticals and nanonutraceuticals research. Curr. Res. Biotechnol..

[122] Zhao C.D., Cheng H., Jiang P.F., Yao Y.J., Han J. (2014). Preparation of lutein loaded particles for improving solubility and stability by Polyvinylpyrrolidone (PVP) as an emulsion-stabilizer. Food Chem..

[123] Dima C., Assadpour E., Dima S., Jafari S.M. (2020). Nutraceutical nanodelivery; An Insight into the bioaccessibility/bioavailability of different bioactive compounds loaded within nanocarriers. Crit. Rev. Food Sci. Nutr..

[124] Dima C., Assadpour E., Dima S., Jafari S.M. (2020). Bioactive-loaded nanocarriers for functional foods: From designing to bioavailability. Curr. Opin. Food Sci..

[125] Leitgeb M., Knez Ž., Primožič M. (2020). Sustainable technologies for liposome preparation. J. Supercrit. Fluids.

[126] Sarangi M., Padhi S. (2018). Novel herbal drug delivery system: An overview. Arch. Med. Health Sci..

[127] Esposto B.S., Jauregi P., Tapia-Blácido D.R., Martelli-Tosi M. (2021). Liposomes vs. chitosomes: Encapsulating food bioactives. Trends Food Sci. Technol..

[128] Sogut O., Aydemir Sezer U., Sezer S. (2021). Liposomal delivery systems for herbal extracts. J. Drug Deliv. Sci. Technol..

[129] Ulrich A.S. (2002). Biophysical aspects of using liposomes as delivery vehicles. Biosci. Rep..

[130] Tan C., Xia S., Xue J., Xie J., Feng B., Zhang X. (2013). Liposomes as Vehicles for Lutein: Preparation, Stability, Liposomal Membrane Dynamics, and Structure. J. Agric. Food Chem..

[131] Tan C., Xue J.S., Abbas S., Feng B., Zhang X., Xia S. (2014). Liposomes as a delivery system for carotenoids: Comparative antioxidant activity of carotenoids as measured by ferric reducing antioxidant power, DPPH assay and lipid peroxidation. J. Agric. Food Chem..

[132] Tan C., Xue J., Lou X.W., Abbas S., Guan Y., Feng B., Zhang X.M., Xia S.Q. (2014). Liposomes as delivery systems for carotenoids: Comparative studies of loading ability, storage stability and in vitro release. Food Funct..

[133] Tan C., Zhang Y., Abbas S., Feng B., Zhang X., Xia S. (2014). Modulation of the carotenoid bioaccessibility through liposomal encapsulation. Colloids Surf. B Biointerfaces.

[134] Xia S., Tan C., Zhang Y., Abbas S., Feng B., Zhang X., Qin F. (2015). Modulating effect of lipid bilayer–carotenoid interactions on the property of liposome encapsulation. Colloids Surf. B Biointerfaces.

[135] Xia F., Hu D., Jin H., Zhao Y., Liang J. (2012). Preparation of lutein proliposomes by supercritical anti-solvent technique. Food Hydrocoll..

[136] Zhao L.S., Temelli F., Curtis J.M., Chen L.Y. (2017). Encapsulation of lutein in liposomes using supercritical carbon dioxide. Food Res. Int..

[137] Trucillo P., Martino M., Reverchon E. (2021). Supercritical Assisted Production of Lutein-Loaded Liposomes and Modelling of Drug Release. Processes.

[138] Tan C., Feng B., Zhang X., Xia W., Xia S. (2016). Biopolymer-coated liposomes by electrostatic adsorption of chitosan (chitosomes) as novel delivery systems for carotenoids. Food Hydrocoll..

[139] Jiao Y., Li D., Liu C., Chang Y., Song J., Xiao Y.J.R.A. (2018). Polypeptide–decorated nanoliposomes as novel delivery systems for lutein. RSC Adv..

[140] Kopec R.E., Gleize B., Borel P., Desmarchelier C., Caris-Veyrat C. (2017). Are lutein, lycopene, and beta-carotene lost through the digestive process?. Food Funct..

[141] Weigel F., Weiss J., Decker E.A., McClements D.J. (2018). Lutein-enriched emulsion-based delivery systems: Influence of emulsifiers and antioxidants on physical and chemical stability. Food Chem..

[142] Lo J.T., Lee T.M., Chen B.H. (2016). Nonionic microemulsions as solubilizers of hydrophobic drugs: Solubilization of paclitaxel. Materials.

[143] McClements D.J. (2012). Nanoemulsions versus microemulsions: Terminology, differences, and similarities. Soft Matter.

[144] Steiner B.M., McClements D.J., Davidov-Pardo G. (2018). Encapsulation systems for lutein: A review. Trends Food Sci Technol..

[145] Vishwanathan R., Wilson T.A., Nicolosi R.J. (2009). Bioavailability of a nanoemulsion of lutein is greater than a lutein supplement. Nano Biomed. Eng..

[146] Frede K., Henze A., Khalil M., Baldermann S., Schweigert F.J., Rawel H. (2014). Stability and cellular uptake of lutein-loaded emulsions. J. Funct. Foods.

[147] Murillo A.G., Aguilar D., Norris G.H., DiMarco D.M., Missimer A., Hu S., Smyth J.A., Gannon S., Blesso C.N., Luo Y. (2016). Compared with powdered lutein, a lutein nanoemulsion increases plasma and liver lutein, protects against hepatic steatosis, and affects lipoprotein metabolism in guinea pigs. J. Nutr..

[148] Kaur I.P., Kakkar S. (2014). Nanotherapy for posterior eye diseases. J. Control. Release.

[149] Reimondez-Troitiño S., Csaba N., Alonso M.J., de la Fuente M. (2015). Nanotherapies for the treatment of ocular diseases. Eur. J. Pharm. Biopharm..

[150] Lim C., Kim D.-W., Sim T., Hoang N.H., Lee J.W., Lee E.S., Youn Y.S., Oh K.T. (2016). Preparation and characterization of a lutein loading nanoemulsion system for ophthalmic eye drops. J. Drug Deliv. Sci. Technol..

[151] Ge Y., Zhang A., Sun R., Xu J.W., Yin T., He H.B., Gou J.X., Kong J., Zhang Y., Tang X. (2020). Penetratin-modified lutein nanoemulsion in-situ gel for the treatment of age-related macular degeneration. Expert Opin. Drug Deliv..

[152] Mehnert W., Mäder K. (2012). Solid lipid nanoparticles: Production, characterization and applications. Adv. Drug Deliv. Rev..

[153] Patil D., Pattewar S., Palival S., Patil G., Sharma S. (2019). Nanostructured lipid carriers: A platform to lipophilic drug for oral bioavailability enhancement. J. Drug Deliv. Ther..

[154] Duong V.A., Nguyen T.T., Maeng H.J. (2020). Preparation of solid lipid nanoparticles and nanostructured lipid carriers for drug delivery and the effects of preparation parameters of solvent injection method. Molecules.

[155] Mitri K., Shegokar R., Gohla S., Anselmi C., Müller R.H. (2011). Lipid nanocarriers for dermal delivery of lutein: Preparation, characterization, stability and performance. Int. J. Pharm..

[156] Shah S., Bhanderi B., Soniwala M., Chavda J. (2021). Lutein-loaded solid lipid nanoparticles for ocular delivery: Statistical optimization and ex vivo evaluation. J. Pharm. Innov..

[157] Tan F., Cui H., Bai C., Qing C., Xu L., Han J. (2021). Preparation, Optimization, and transcorneal permeability study of Lutein-loaded Solid Lipid Nanoparticles. J. Drug Deliv. Sci. Technol..

[158] Lacatusu I., Mitrea E., Badea N., Stan R., Oprea O., Meghea A. (2013). Lipid nanoparticles based on omega-3 fatty acids as effective carriers for lutein delivery. Preparation and in vitro characterization studies. J. Funct. Foods.

[159] Liu C.H., Chiu H.C., Wu W.C., Sahoo S.L., Hsu C.Y. (2014). Novel lutein loaded lipid nanoparticles on porcine corneal distribution. J. Ophthalmol..

[160] Mahapatro A., Singh D.K. (2011). Biodegradable nanoparticles are excellent vehicle for site directed in vivo delivery of drugs and vaccines. J. Nanobiotechnol..

[161] Begines B., Ortiz T., Pérez-Aranda M., Martínez G., Merinero M., Argüelles-Arias F., Alcudia A. (2020). Polymeric nanoparticles for drug delivery: Recent developments and future prospects. Nanomaterials.

[162] Goldberg M., Langer R., Xinqiao J. (2007). Nanostructured materials for applications in drug delivery and tissue engineering. J. Biomat. Sci. Polym. E.

[163] Jiménez-Gómez C.P., Cecilia J.A. (2020). Chitosan: A natural biopolymer with a wide and varied range of applications. Molecules.

[164] Pathomthongtaweechai N., Muanprasat C. (2021). Potential applications of chitosan-based nanomaterials to surpass the gastrointestinal physiological obstacles and enhance the intestinal drug absorption. Pharmaceutics.

[165] Mikušová V., Mikuš P. (2021). Advances in chitosan-based nanoparticles for drug delivery. Int. J. Mol. Sci..

[166] Hong D.Y., Lee J.-S., Lee H.G. (2016). Chitosan/poly-γ-glutamic acid nanoparticles improve the solubility of lutein. Int. J. Biol. Macromol..

[167] Chaiyasan W., Srinivas S.P., Tiyaboonchai W. (2015). Crosslinked chitosan-dextran sulfate nanoparticle for improved topical ocular drug delivery. Mol. Vis..

[168] Arunkumar R., Prashanth K.V.H., Baskaran V. (2013). Promising interaction between nanoencapsulated lutein with low molecular weight chitosan: Characterization and bioavailability of lutein in vitro and in vivo. Food Chem..

[169] Makadia H.K., Siegel S.J. (2011). Poly Lactic-*co*-Glycolic Acid (PLGA) as Biodegradable Controlled Drug Delivery Carrier. Polymers.

[170] Mundargi R., Babu V., Rangaswamy V., Patel P., Aminabhavi T. (2008). Nano/micro technologies for delivering macromolecular therapeutics using poly(D,L-lactide-co-glycolide) and its derivatives. J. Control. Release.

[171] Kamil A., Smith D.E., Blumberg J.B., Astete C., Sabliov C., Chen C.Y.O. (2016). Bioavailability and biodistribution of nanodelivered lutein. Food Chem..

[172] Arunkumar R., Prashanth K.V., Manabe Y., Hirata T., Sugawara T., Dharmesh S.M., Baskaran V. (2015). Biodegradable poly(lactic-co-glycolic acid)-polyethylene glycol nanocapsules: An efficient carrier for improved solubility, bioavailability, and anticancer property of lutein. J. Pharm. Sci..

[173] Bolla P.K., Gote V., Singh M., Patel M., Clark B.A., Renukuntla J. (2020). Lutein-loaded, biotin-decorated polymeric nanoparticles enhance lutein uptake in retinal cells. Pharmaceutics.

[174] Bolla P.K., Gote V., Singh M., Yellepeddi V.K., Patel M., Pal D., Gong X., Sambalingam D., Renukuntla J. (2020). Preparation and characterization of lutein loaded folate conjugated polymeric nanoparticles. J. Microencapsul..

[175] Chittasupho C., Posritong P., Ariyawong P. (2018). Stability, Cytotoxicity, and Retinal Pigment Epithelial Cell Binding of Hyaluronic Acid-Coated PLGA Nanoparticles Encapsulating Lutein. AAPS Pharmscitech.

[176] Dhas N., Mehta T. (2020). Cationic biopolymer functionalized nanoparticles encapsulating lutein to attenuate oxidative stress in effective treatment of Alzheimer’s disease: A non-invasive approach. Int. J. Pharm..

[177] Reboul E. (2019). Mechanisms of carotenoid intestinal absorption: Where do we stand?. Nutrients.

[178] Yao Y., Lin J.J., Chee X.Y.J., Liu M.H., Khan S.A., Kim J.E. (2021). Encapsulation of lutein via microfluidic technology: Evaluation of stability and in vitro bioaccessibility. Foods.

[179] Ranganathan A., Hindupur R., Vallikannan B. (2016). Biocompatible lutein-polymer-lipid nanocapsules: Acute and subacute toxicity and bioavailability in mice. Mater. Sci. Eng. C.

[180] Toragall V., Baskaran V. (2021). Chitosan-sodium alginate-fatty acid nanocarrier system: Lutein bioavailability, absorption pharmacokinetics in diabetic rat and protection of retinal cells against H_2_O_2_ induced oxidative stress in vitro. Carbohydr. Polym..

[181] Ranganathan A., Manabe Y., Sugawara T., Hirata T., Shivanna N., & Baskaran V. (2019). Poly(D,L-lactide-co-glycolide)-phospholipid nanocarrier for efficient delivery of macular pigment lutein: Absorption pharmacokinetics in mice and antiproliferative effect in Hep G2 cells. Drug Deliv. Transl. Res..

[182] Toragall V., Jayapala N., Vallikannan B. (2020). Chitosan-oleic acid-sodium alginate a hybrid nanocarrier as an efficient delivery system for enhancement of lutein stability and bioavailability. Int. J. Biol. Macromol..

[183] Shwetha H.J., Shilpa S., Mukherjee M.B., Ambedkar R., Raichur A.M., Lakshminarayana R. (2020). Fabrication of chitosan nanoparticles with phosphatidylcholine for improved sustain release, basolateral secretion, and transport of lutein in Caco-2 cells. Int. J. Biol. Macromol..

